# Prognostic value of lymphopenia in early breast cancer: learnings from the prospective CANTO cohort

**DOI:** 10.1186/s13058-026-02315-7

**Published:** 2026-06-09

**Authors:** Stéphane Vignot, Youenn Drouet, Antonio Di Meglio, Anne-Laure Martin, Catherine Gaudin, Aurélie Bertaut, Marion Fournier, William Jacot, Claire Cheymol, Anne Kieffer, Carole Tarpin, Florence Lerebours, Christophe Caux, Sandrine Boyault, Christine Ménétrier-Caux, Olivier Trédan

**Affiliations:** 1 IRMAIC, université Reims Champagne Ardenne, 1, rue du Maréchal Juin, 51100 Reims, France; 2 Medical Oncology Department, Institut Godinot, 1, rue du Général Koenig, 51100 Reims, France; 3 Prevention and Public Health Department, Centre Léon Bérard, 69008 Lyon, France; 4 CANTO Breast scientific coordinator, Cancer Survivorship Program, INSERM 981, Gustave Roussy, Villejuif, France; 5 Data and Partnerships Department, Unicancer, Paris, France; 6 Methodology and Biostatistics Unit, Centre Georges-François Leclerc, Dijon, France; 7 Surgery and Oncology Department, Institut Bergonié, Bordeaux, France; 8 Medical Oncology Department, Institut du Cancer de Montpellier, Université de Montpellier, INSERM U1194, Montpellier, France; 9 Medical Oncology Department, Centre Oscar Lambret, Lille, France; 10 Medical Oncology Department, Institut de cancérologie de Lorraine, Vandoeuvre-Lès-Nancy, France; 11 Medical Oncology Department, Institut Paoli Calmettes, Marseille, France; 12 Medical Oncology Department, Institut Curie-site de Saint-Cloud, Saint-Cloud, France; 13 Cancer Research Center of Lyon, INSERM 1052 - CNRS 5286, Centre Léon Bérard, Université de Lyon, Université Claude Bernard Lyon 1, 69008 Lyon, France; 14 Cancer Genomic Platform, Cancer Research Center of Lyon, Centre Léon Bérard, CNRS, INSERM, Université Lyon 1, 69008 Lyon, France; 15 Medical Oncology Department, Centre Léon Bérard, 69008 Lyon, France

**Keywords:** Early Breast cancer, Lymphopenia, Prognosis, Relapse risk

## Abstract

**Background:**

Lymphopenia has been associated with poor outcomes in metastatic breast cancer (BC), but its prognostic relevance in early-stage disease remains unclear. This study aimed to evaluate the prognostic value of baseline lymphopenia in patients with non-metastatic BC using data from the prospective French CANTO cohort (NCT01993498).

**Methods:**

10,854 patients with baseline absolute lymphocyte count (ALC) were analysed. Lymphopenia was defined as ALC < 1.0 × 10⁹ cells/L, measured before any cancer treatment. Relapse-free survival (RFS), overall survival (OS), and treatment-related toxicities were assessed using Kaplan-Meier estimates, piecewise-Cox regression models, and cause-specific hazard analyses considering pre-specified time periods.

**Results:**

Baseline lymphopenia was observed in 342 patients (3.1%). It was associated with ECOG performance status > 0, hypoalbuminemia, and prior neoplasia. Lymphopenia correlated with inferior 5-year RFS (89.1% vs. 92.9%) and OS (92.5% vs. 96.4%). In multivariable analyses, lymphopenia was associated with increased risk of early relapse during the first 24 months (HR = 2.11; 95%CI: 1.28–3.48; p = 0.003), but not beyond, and no independent prognostic impact on OS was observed. No significant differences were found in surgical complications or chemotherapy dose intensity, though granulocyte colony-stimulating factor use was higher among lymphopenic patients.

**Conclusion:**

Baseline lymphopenia is uncommon in early BC but may serve as a marker of early relapse risk. Although it does not independently predict long-term survival, its presence could reflect underlying tumour aggressiveness or patient frailty. These findings support further investigation of lymphocyte subpopulations to refine prognostic stratification in early BC.

**Trial registration:**

Approved by French ethics committee in 2011 (ID-RCB:2011-A01095- 36,11–039 /NCT01993498 [20FEB2012]).

**Supplementary Information:**

The online version contains supplementary material available at 10.1186/s13058-026-02315-7.

## Introduction

Breast cancer (BC) continues to be a significant health challenge for women globally. Recent progress in early detection and treatment options have improved the prognosis. Most patients are now diagnosed at localized stages, making the decision of whether to use adjuvant medical treatments to reduce recurrence risk a critical issue in clinical practice. The prognostic factors guiding these decisions are well-established and have been further refined by molecular signatures [1]. In this context, tumor characteristics are exclusively considered to estimate prognosis, independently of the patient’s profile. The appropriateness of the proposed treatments must then be assessed concerning the clinical context (including Eastern Cooperative Oncology Group performance status (ECOG-PS), age, comorbidities, and oncogeriatric assessment if relevant), while also considering the patient’s preferences. It is important to incorporate non-tumoral prognostic factors into the decision-making process for adjuvant chemotherapy, as this would add a valuable element to the development of personalized medicine. In metastatic setting, tumour-independent prognostic factors are used to better estimate the prognosis. Lymphopenia (i.e. absolute lymphocyte count (ALC) *≤* 1.0 × 10^9^ cells/L) serves as an example of this approach. Indeed, lymphocytes play a crucial role in the immune system’s ability to recognize and eliminate cancer cells [2]. Several studies have suggested that lymphopenia before treatment may act as an independent prognostic indicator in patients with BC in metastatic setting. Patients with lower ALC tend to have a poorer prognosis, including shorter relapse-free survival (RFS) and overall survival (OS) rates [3–5]. Therefore, the prognostic value of lymphopenia merits further investigation in patients with early BC, given that lymphopenia may reflect both tumour aggressiveness and patient frailty.

In this study, we assess the prognostic value of lymphopenia by analysing observational data collected prospectively in the CANTO cohort. CANcer TOxicities (CANTO) NCT01993498 / 2011-A01095-36 is a French prospective study with the objective of identifying predictive factors of acute and chronic toxicities in patients treated for stage I–III BC [6].This large and ongoing study allows access to a large set of clinical and biological data for more than 10,000 BC patients with long-term follow-up. This offers a unique opportunity to assess the putative additional value of lymphopenia in estimating the prognosis of early BC.

## Patients and methods

### CANTO cohort

CANTO is a French longitudinal prospective multicentre cohort study of women with stage I–IIIA BC treated at one of the 26 participating French cancer centres. CANTO collected data at diagnosis of BC (i.e., before initiation of any cancer treatment) and then at different time point after diagnosis. Enrolment started on March 20, 2012. Details on inclusion criteria and collected data have been previously reported [6]. CANTO includes detailed information on treatments received. In this cohort, 73% underwent conservative surgery and 27% mastectomy. Regarding chemotherapy, 46% received no chemotherapy, 13% neo-adjuvant chemotherapy, 40% adjuvant chemotherapy, and 0.4% both. Radiotherapy was administered in 91% of patients, consistent with standard early breast cancer management. The study was approved by the ethics committee (ID-RCB:2011-A01095-36). All patients enrolled in the study provide written informed consent.

### Baseline lymphopenia

ALC was measured at baseline before any treatment, during the first biological assessment following BC diagnosis. Based on prior clinical and epidemiological studies, ALC < 1.0 × 10⁹ cells/L was pre-specified as the primary definition of lymphopenia. Two additional thresholds(< 1.5 × 10⁹ cells/L and < 0.7 × 10⁹ cells/L) previously used to characterize severity [7, 8] were evaluated secondarily to assess the robustness of this prespecified cut-off. Survival patterns were broadly similar across these thresholds (Figure S1), supporting the use of ALC < 1.0 × 10⁹ cells/L for the main analyses.

### Survival and toxicity outcomes

The prognostic value of lymphopenia was estimated for OS, defined as the time elapsed from baseline biological assessment to death from any cause or latest news, and for RFS, defined as the time until death, loco‑regional recurrence, distant recurrence, or contralateral invasive breast cancer, whichever comes first. For the cause‑specific analyses, clinical event definitions were as follows: relapse included any documented loco‑regional or distant recurrence or contralateral invasive breast cancer; death without relapse was defined as death from any cause occurring before any documented relapse; and death after relapse as death occurring after a documented relapse event. OS and RFS were analysed in the following three pre-specified time-periods: (i) the firsts 24 months, which embedded the treatment period; (ii) between 24 and 60 months, which is recognized critical for the risk of relapse [9, 10] and (iii) after 60 months. This allowed us to investigate the relation between lymphopenia and OS and RFS, as well as others factors, in three key periods of the patient’s follow-up, and a way to extend the Cox model accounting for non-proportional hazard (i.e., time-varying effects) (see Statistical Analyses section). The relation between baseline lymphopenia and toxicity incidence was also analysed with a focus on acute toxicities and dose-intensity of chemotherapy treatment (planned vs. delivered).

### Statistical analyses

Missing data were handled using multiple imputation [11], assuming data was “Missing At Random”, and generating 30 complete datasets using the missMDA R package [12]. Of note, survival event indicator and the Nelson–Aalen estimator of the hazard function were both included in the imputation model, but were not imputed [13].

Survival curves were drawn with the Kaplan-Meier method. Multivariable survival regression models were developed using the piecewise-Cox approach, extending classical Cox regression to non-proportionality (i.e. including time-varying coefficients) [14]. The Schoenfeld residuals were used to examine graphically and test the violation of the proportionality assumption, in both Cox and Piecewise-Cox models. All models included the following pre-specified covariables: lymphocyte count, age at diagnosis, ECOG-PS scale, Charlson Comorbidity Index, Body Mass Index (BMI), albuminemia, smoking history, history of cancer, TNM tumor staging, tumor histology, tumor subtype, tumor grade and Mitotic Index (MI). All two-by-two interactions between covariables and lymphocyte count were tested using the Benjamini and Hochberg method as correction for multiple testing. Specific interactions between lymphopenia and (i) BC stage, and (ii) the triple-negative (TNBC) subtype were also tested formally using likelihood ratio tests. Multicollinearity was assessed using the Generalized Variance-Inflation Factor.

Competing risk analyses were implemented to evaluate the association between baseline lymphopenia and the occurrence of relapse, death without relapse, and death after relapse. We used cause-specific hazard models, which estimate the instantaneous risk of a given event in the presence of competing events by censoring individuals at the time of the competing outcome [15]. This approach allowed us to separately model the hazard functions for relapse, death without relapse, and death after relapse, while accounting for the fact that these events preclude one another. Multivariable cause-specific Cox regressions were performed using the same prespecified covariates as in the overall survival models, ensuring consistency across analyses. Event-specific hazard ratios (HRs) were derived, and cumulative incidence functions were estimated to illustrate the absolute risk of each event type over time. This dual approach provided complementary insights into how lymphopenia at diagnosis may differentially influence distinct survival outcomes. All analyses were performed using R (version 4.4.1, R Foundation for Statistical Computing, Vienna, Austria). A p-value < 0.05 was considered statistically significant.

## Results

### Cohort description

**Fig. 1:**
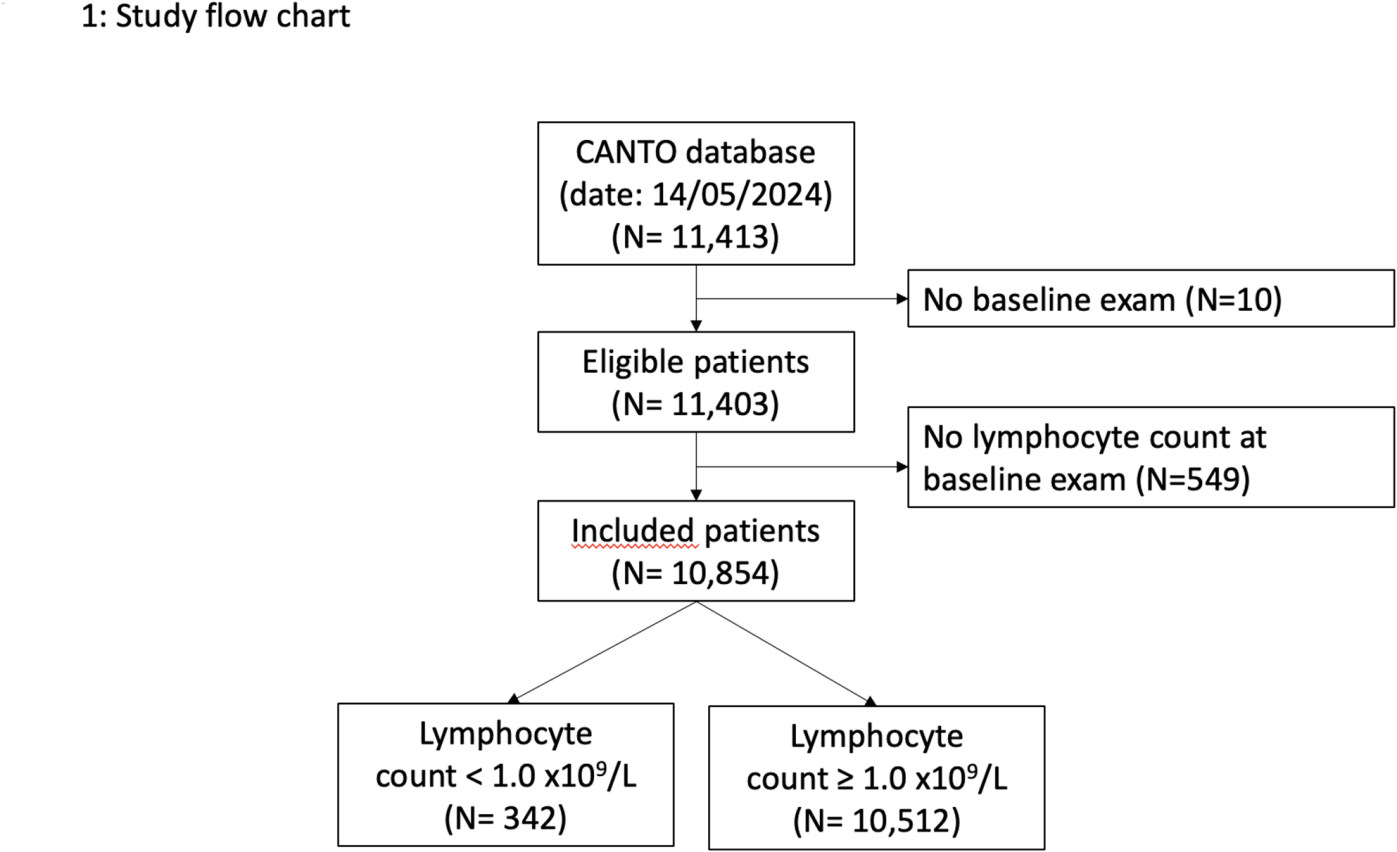
Study flow chart

Among the 11,413 patients included in CANTO study in May 2024, lymphocyte count at baseline was available for 10,854 patients who were included in the analysis, with a median follow-up of 7.04 years (Fig. 1).

At baseline, 2,436 BC patients (22.4%) presented ALC < 1.5 × 10^9^ cells/L. Lymphopenia defined by ALC < 1.0 × 10^9^ cells/L was detected in 342 patients (3.1%) of including 85 patients (0.78%) with ALC < 0.7 × 10^9^ cells/L / µL. Patients’ characteristics are presented in Table 1. Lymphopenia was significantly more frequent for ECOG-PS > 0 patients, patients with hypoalbuminemia and for patients with personal history of previous neoplasia. Lymphopenia was less frequent for obese patients and active smokers. No association was observed with the Charlson Comorbidity Index.

**Table 1:** Patient and Tumour characteristics according to lymphopenia measured at baseline

	Lymphocyte count at baseline			
	< 1.0 × 10^9^ /L (*N* = 342)	≥ 1.0 × 10^9^ /L (*N* = 10,512)	Total (*N* = 10,854)	*P* value
Patient characteristics at baseline				
**Age at diagnostic (years)**				0.496
Missing	1	23	24	
Mean (SD)	56.38 (12.52)	55.95 (11.29)	55.97 (11.33)	
Range	22.00–88.00	20.00–89.00	20.00–89.00	
				0.086
18—39 years	31 (9.1%)	898 (8.6%)	929 (8.6%)	
40—49 years	90 (26.4%)	2,713 (25.9%)	2,803 (25.9%)	
50—59 years	97 (28.4%)	2,987 (28.5%)	3,084 (28.5%)	
60—69 years	77 (22.6%)	2,883 (27.5%)	2,960 (27.3%)	
≥70 years	46 (13.5%)	1,008 (9.6%)	1,054 (9.7%)	
**Menopausal Status**				0.135
Missing	7	163	170	
Pre menopause	141 (42.1%)	3,939 (38.1%)	4,080 (38.2%)	
Post menopause	194 (57.9%)	6,410 (61.9%)	6,604 (61.8%)	
**ECOG-PS**				**0.003**
Missing	34	993	1027	
ECOG 0	279 (90.6%)	8,999 (94.5%)	9,278 (94.4%)	
ECOG > 0	29 (9.4%)	520 (5.5%)	549 (5.6%)	
**Charlson Comorbidity Index**				0.14
Missing	19	1028	1047	
0 points	260 (80.5%)	7,616 (80.3%)	7,876 (80.3%)	
1 points	35 (10.8%)	1,114 (11.7%)	1,149 (11.7%)	
2 points	13 (4.0%)	499 (5.3%)	512 (5.2%)	
≥ 3 points	15 (4.6%)	255 (2.7%)	270 (2.8%)	
**Body Mass Index (BMI)**				**< 0.001**
Missing	1	43	44	
Mean (SD)	24.70 (5.10)	25.96 (5.38)	25.92 (5.38)	
Range	14.80–49.50	14.70–59.00	14.70–59.00	
				**< 0.001**
underweight (< 18.5)	11 (3.2%)	258 (2.5%)	269 (2.5%)	
normal weight (18.5—24.9)	212 (62.2%)	5,130 (49.0%)	5,342 (49.4%)	
overweight (25—29.9)	71 (20.8%)	3,008 (28.7%)	3,079 (28.5%)	
obesity (≥ 30)	47 (13.8%)	2,073 (19.8%)	2,120 (19.6%)	
**Albuminemia**				**0.018**
Missing	72	2,084	2,156	
Mean (SD)	42.30 (4.49)	42.84 (3.63)	42.82 (3.66)	
Range	7.00–52.00	4.80–59.00	4.80–59.00	
**Smoking history**				**< 0.001**
Missing	7	149	156	
Never smoker	222 (66.3%)	6,168 (59.5%)	6,390 (59.7%)	
Former smoker	84 (25.1%)	2,289 (22.1%)	2,373 (22.2%)	
Current smoker	29 (8.7%)	1,906 (18.4%)	1,935 (18.1%)	
**History of previous neoplasia**				**0.034**
Missing	4	140	144	
No	301 (89.1%)	9,564 (92.2%)	9,865 (92.1%)	
Yes	37 (10.9%)	808 (7.8%)	845 (7.9%)	
***Tumour characteristics***				
**Stage**				0.074
Missing	10	130	140	
0/I	160 (48.2%)	5,022 (48.4%)	5,182 (48.4%)	
II	128 (38.6%)	4,359 (42.0%)	4,487 (41.9%)	
III	44 (13.3%)	1,001 (9.6%)	1,045 (9.8%)	
**Histology**				0.496
Missing	2	19	21	
Invasive ductal carcinoma	271 (79.7%)	8,156 (77.7%)	8,427 (77.8%)	
Invasive lobular carcinoma	43 (12.6%)	1,337 (12.7%)	1,380 (12.7%)	
Other	26 (7.6%)	1,000 (9.5%)	1,026 (9.5%)	
**Subtype**				**0.041**
Missing	5	71	76	
HR+/HER2-	242 (71.8%)	8,032 (76.9%)	8,274 (76.8%)	
HR+/HER2+	32 (9.5%)	1,014 (9.7%)	1,046 (9.7%)	
HR-/HER2+	16 (4.7%)	388 (3.7%)	404 (3.7%)	
HR-/HER2-	47 (13.9%)	1,007 (9.6%)	1,054 (9.8%)	
**Grade**				0.717
Missing	8	109	117	
1	63 (18.9%)	1,807 (17.4%)	1,870 (17.4%)	
2	172 (51.5%)	5,559 (53.4%)	5,731 (53.4%)	
3	99 (29.6%)	3,037 (29.2%)	3,136 (29.2%)	
**Mitotic Index**				0.412
Missing	19	269	288	
1	190 (58.8%)	5,927 (57.9%)	6,117 (57.9%)	
2	80 (24.8%)	2,350 (22.9%)	2,430 (23.0%)	
3	53 (16.4%)	1,966 (19.2%)	2,019 (19.1%)	

HR+: hormonal receptors expression (either estrogen or progesterone) above 10%; HER2+: overexpression of HER2 (HER2 3 + or HER2 2 + FISH+); CI : confidence interval;

Regarding tumour characteristics, the TNBC subtype was slightly more frequent in patients with lymphopenia (13.9% of lymphopenic patients vs. 9.6% without lymphopenia). No statistically significant association was detected considering other classic tumour prognostic factors used for adjuvant therapy decision-making (stage, grade, mitotic index).

### Survival analysis according to baseline lymphopenia

**Fig. 2:**
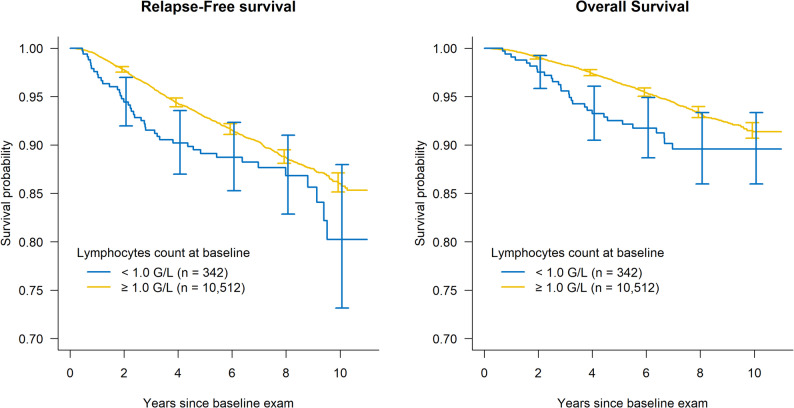
Unadjusted analyses for RFS and OS according to lymphopenia

Survival outcomes according to lymphopenia measured at baseline are presented in Table 2 and Kaplan-Meier survival curves for RFS and OS according to lymphopenia are presented in Fig. 2. Lymphopenia was associated with lower RFS (5-year RFS rates 89.1% vs. 92.9%, log-rank test: *P* = 0.050) and lower OS (5-year OS rates 92.5% vs. 96.4%, log-rank test: *P* = 0.01).

**Table 2:** Survival outcomes according to lymphopenia measured at baseline

	Lymphocyte count at baseline		
	< 1.0 × 10^9^ /L (*N* = 342)	≥ 1.0 × 10^9^ /L (*N* = 10,512)	Total (*N* = 10,854)
Follow-up (years)			
Median (95% CI)	7.06 (6.65–7.32)	7.04 (6.98–7.11)	7.04 (6.98–7.11)
**Relapse-free survival (RFS)**			
Number of events	42	1,002	1,044
2-year RFS rate (95% CI)	94.4% (92.0–97.0)	97.8% (97.5–98.1)	97.7% (97.4–98.0)
5-year RFS rate (95% CI)	89.1% (85.7–92.7)	92.9% (92.4–93.4)	92.8% (92.3–93.3)
**Overall survival (OS)**			
Number of events	29	576	605
2-year OS rate (95% CI)	97.5% (95.8–99.2)	99.0% (98.8–99.2)	99.0% (98.8–99.2)
5-year OS rate (95% CI)	92.5% (89.6–95.5)	96.4% (96.0–96.8)	96.3% (95.9–96.7)

The prognostic value of lymphopenia on RFS was explored using piecewise-Cox multivariable regression models considering the following pre-specified time periods P1, P2 and P3 (Table 3). This approach was used to address time‑varying effects identified through assessment of the proportional hazards assumption using Schoenfeld residuals as detailed in Methods. Lymphopenia was associated with lower RFS only within P1 with HR of 2.11 (95%CI: 1.28–3.48; *P* = 0.003). The other factors associated with lower RFS within P1 were: age at diagnosis ≥ 70 years, BMI < 18.5, tumour stage, and the TNBC subtype. Lymphopenia was not associated with lower RFS in P2 and P3. Factors associated with lower RFS in both P2 and P3 were: age at diagnosis, smoking history, history of previous cancer, tumour stage, tumour grade and mitotic index.

**Table 3:** Relapse-free survival (RFS) analysis according to time elapsed from diagnosis

Variable name		HR for Relapse-Free Survival (95%CI)		
	Value	Time period P1: 0–24 m	Time period P2: 24–60 m	Time period P3: 60 m+
**Lymphocyte count**	< 1.0 × 10^9^ /L (ref ≥ 1.0 × 10^9^ /L)	**2.110 (1.28—3.48;** *p*** = 0.003)**	1.110 (0.67—1.84; *p* = 0.685)	0.786 (0.40—1.55; *p* = 0.488)
**Age at diagnosis**	18—39 years	1.304 (0.86—1.97; *p* = 0.205)	1.345 (0.98—1.85; *p* = 0.066)	**1.784 (1.18—2.70;** *p*** = 0.006)**
	40—49 years	0.936 (0.66—1.34; *p* = 0.714)	0.848 (0.65—1.10; *p* = 0.214)	1.262 (0.91—1.75; *p* = 0.165)
	50—59 years	(reference)		
	60—69 years	1.056 (0.72—1.55; *p* = 0.779)	1.111 (0.86—1.44; *p* = 0.429)	1.360 (0.98—1.89; *p* = 0.066)
	≥ 70 years	**1.775 (1.12—2.81;** *p*** = 0.015)**	**1.651 (1.20—2.27;** *p*** = 0.002)**	**3.253 (2.26—4.68;** *p*** < 0.001)**
**ECOG-PS**	ECOG > 0 (ref 0)	1.589 (0.98—2.57; *p* = 0.060)	0.757 (0.48—1.19; *p* = 0.230)	1.149 (0.73—1.82; *p* = 0.554)
**Charlson Comorbidity Index**	CCI > 0 (ref 0)	1.008 (0.71—1.44; *p* = 0.963)	1.226 (0.97—1.55; *p* = 0.087)	0.970 (0.72—1.30; *p* = 0.840)
**BMI**	underweight (< 18.5)	**2.097 (1.10—3.99;** *p*** = 0.024)**	1.475 (0.85—2.55; *p* = 0.163)	0.822 (0.36—1.89; *p* = 0.644)
	normal weight (18.5—24.9)	(reference)		
	overweight (25—29.9)	0.946 (0.69—1.29; *p* = 0.728)	1.047 (0.84—1.30; *p* = 0.679)	1.147 (0.88—1.49; *p* = 0.300)
	obesity (≥ 30)	1.095 (0.79—1.52; *p* = 0.588)	1.144 (0.90—1.45; *p* = 0.270)	1.157 (0.86—1.56; *p* = 0.337)
**Albuminemia**	numeric value	0.982 (0.95—1.02; *p* = 0.313)	0.993 (0.97—1.02; *p* = 0.576)	0.992 (0.96—1.02; *p* = 0.621)
**Smoking history**	Never_smoker	(reference)		
	Former_smoker	1.066 (0.76—1.49; *p* = 0.709)	1.168 (0.93—1.46; *p* = 0.180)	1.038 (0.77—1.39; *p* = 0.802)
	Current_smoker	1.265 (0.91—1.77; *p* = 0.167)	**1.335 (1.04—1.71;** *p*** = 0.021)**	**1.627 (1.21—2.18;** *p*** = 0.001)**
**History of cancer**	Yes (reference No)	0.744 (0.43—1.29; *p* = 0.292)	**1.533 (1.13—2.07;** *p*** = 0.005)**	**1.470 (1.01—2.13;** *p*** = 0.042)**
**Stage**	0/I	(reference)		
	II	**2.939 (2.02—4.28;** *p*** < 0.001)**	**2.297 (1.81—2.92;** *p*** < 0.001)**	**1.468 (1.15—1.88;** *p*** = 0.002)**
	III	**7.601 (5.02—11.51;** *p*** < 0.001)**	**6.440 (4.96—8.36;** *p*** < 0.001)**	**2.652 (1.90—3.70;** *p*** < 0.001)**
**Histology**	Invasive ductal carcinoma	(reference)		
	Invasive lobular carcinoma	0.827 (0.49—1.40; *p* = 0.477)	0.984 (0.74—1.31; *p* = 0.913)	**1.379 (1.01—1.89;** *p*** = 0.045)**
	Other	1.002 (0.66—1.53; *p* = 0.993)	0.885 (0.64—1.21; *p* = 0.449)	1.136 (0.78—1.65; *p* = 0.501)
**Subtype**	HR+/HER2-	(reference)		
	HR+/HER2+	0.617 (0.34—1.13; *p* = 0.119)	0.836 (0.61—1.14; *p* = 0.256)	0.775 (0.51—1.17; *p* = 0.220)
	HR-/HER2+	1.725 (0.97—3.08; *p* = 0.065)	0.727 (0.45—1.18; *p* = 0.197)	0.513 (0.25—1.05; *p* = 0.068)
	HR-/HER2-	**4.841 (3.42—6.86;** *p*** < 0.001)**	1.174 (0.86—1.59; *p* = 0.306)	0.993 (0.65—1.51; *p* = 0.975)
**Grade**	1	(reference)		
	2	1.144 (0.61—2.15; *p* = 0.675)	**1.956 (1.28—2.99;** *p*** = 0.002)**	**1.618 (1.10—2.38;** *p*** = 0.015)**
	3	1.829 (0.81—4.12; *p* = 0.145)	**2.626 (1.57—4.40;** *p*** < 0.001)**	1.148 (0.64—2.07; *p* = 0.645)
**Mitotic Index**	1	(reference)		
	2	1.314 (0.81—2.12; *p* = 0.265)	**1.404 (1.07—1.84;** *p*** = 0.014)**	1.145 (0.82—1.59; *p* = 0.419)
	3	1.337 (0.76—2.36; *p* = 0.317)	1.236 (0.85—1.81; *p* = 0.273)	**1.875 (1.13—3.12;** *p*** = 0.016)**

**Fig. 3:**
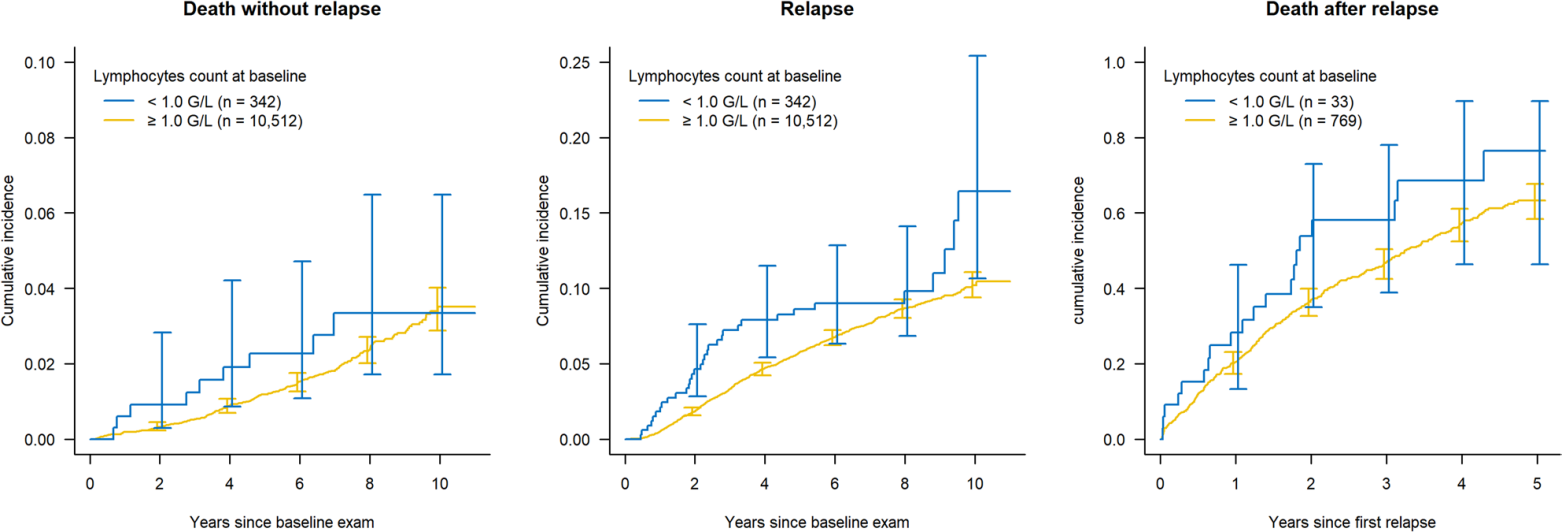
Cumulative incidence of death without relapse, relapse, and death after relapse according to baseline lymphopenia

Cause-specific hazards for relapse, death without relapse and death after relapse were then explored within the three time periods using competitive risk modelling (Fig. 3; Table 4). Lymphopenia < 1.0 × 10^9^ cells/L was associated with higher risk of early relapse within P1 with a HR of 2.049 (95%CI: 1.17–3.58; *P* = 0.012). The known tumoral prognostic factors were also found associated with higher risk of early relapse within P1: stage II and III, triple-negative phenotype and tumour grade > 1. Patients with baseline lymphopenia also had higher rates of death without relapse and death after relapse (Fig. 3), but without reaching statistical significance in the multivariable analysis (Table 4). Lymphopenia was not associated with higher risk of relapse within the P2 and P3 time periods.

**Table 4:** Cause-specific hazard modeling of relapse, death without relapse and death after relapse

Variable Name	Value	HR for Relapse (95%CI)			HR for Death Without Relapse (95%CI)			HR for Death After Relapse (95%CI)	
		Time period: 0–24 m	Time period: 24–60 m	Time period: 60 m+	Time period: 0–24 m	Time period: 24–60 m	Time period: 60 m+	Time period: 0–24 m	Time period: 24 m+
**Lymphocyte count**	< 1.0 × 10^9^ /L (ref ≥ 1.0 × 10^9^ /L)	**2.049 (1.17—3.58;** *p*** = 0.012)**	1.023 (0.57—1.83; *p* = 0.938)	0.825 (0.37—1.83; *p* = 0.636)	2.625 (0.76—9.10; *p* = 0.128)	1.701 (0.63—4.61; *p* = 0.296)	0.674 (0.15—2.96; *p* = 0.601)	0.952 (0.54—1.69; *p* = 0.867)	0.714 (0.19—2.72; *p* = 0.620)
**Age at diagnosis**	18—39 years	1.381 (0.90—2.11; *p* = 0.137)	**1.477 (1.06—2.06;** *p*** = 0.022)**	**2.163 (1.37—3.42;** *p*** = 0.001)**	NE	0.479 (0.14—1.67; *p* = 0.249)	0.621 (0.18—2.17; *p* = 0.455)	**0.512 (0.33—0.78;** *p*** = 0.002)**	0.783 (0.42—1.44; *p* = 0.431)
	40—49 years	0.930 (0.64—1.36; *p* = 0.708)	0.971 (0.74—1.28; *p* = 0.833)	1.451 (1.00—2.11; *p* = 0.052)	0.957 (0.30—3.08; *p* = 0.941)	NE	0.711 (0.32—1.59; *p* = 0.407)	0.892 (0.64—1.24; *p* = 0.498)	0.890 (0.52—1.53; *p* = 0.674)
	50—59 years	(reference)			(reference)			(reference)	
	60—69 years	0.906 (0.59—1.40; *p* = 0.656)	0.913 (0.67—1.24; *p* = 0.563)	1.174 (0.79—1.75; *p* = 0.427)	2.368 (0.89—6.30; *p* = 0.084)	**2.172 (1.25—3.78;** *p*** = 0.006)**	**2.104 (1.12—3.94;** *p*** = 0.020)**	0.978 (0.68—1.40; *p* = 0.904)	0.897 (0.49—1.65; *p* = 0.726)
	≥ 70 years	1.114 (0.62—2.01; *p* = 0.719)	1.163 (0.78—1.74; *p* = 0.462)	1.385 (0.81—2.38; *p* = 0.237)	**7.457 (2.87—19.36;** *p*** < 0.001)**	**4.199 (2.31—7.65;** *p*** < 0.001)**	**10.67 (5.73—19.87;** *p*** < 0.001)**	**1.744 (1.06—2.88;** *p*** = 0.030)**	1.467 (0.75—2.87; *p* = 0.261)
**ECOG-PS**	ECOG > 0 (ref 0)	1.055 (0.55—2.03; *p* = 0.872)	0.653 (0.37—1.15; *p* = 0.141)	0.744 (0.36—1.55; *p* = 0.430)	**4.053 (1.84—8.92;** *p*** < 0.001)**	0.826 (0.34—2.01; *p* = 0.674)	**1.896 (1.05—3.42;** *p*** = 0.034)**	1.069 (0.57—1.99; *p* = 0.834)	1.423 (0.52—3.93; *p* = 0.494)
**Charlson Comorbidity Index**	CCI > 0 (ref 0)	0.988 (0.66—1.48; *p* = 0.955)	1.199 (0.92—1.57; *p* = 0.182)	0.992 (0.69—1.43; *p* = 0.967)	1.115 (0.52—2.39; *p* = 0.779)	1.412 (0.86—2.32; *p* = 0.173)	0.929 (0.56—1.53; *p* = 0.774)	**1.389 (1.00—1.93;** *p*** = 0.049)**	1.053 (0.62—1.78; *p* = 0.845)
**BMI**	underweight (< 18.5)	1.979 (0.98—4.02; *p* = 0.059)	1.115 (0.56—2.20; *p* = 0.754)	0.515 (0.16—1.66; *p* = 0.265)	3.337 (0.77—14.43; *p* = 0.107)	**3.448 (1.16—10.22;** *p*** = 0.026)**	1.587 (0.40—6.32; *p* = 0.512)	**1.929 (1.04—3.59;** *p*** = 0.038)**	1.378 (0.19—9.86; *p* = 0.749)
	normal weight (18.5—24.9)	(reference)			(reference)			(reference)	
	overweight (25—29.9)	1.061 (0.73—1.53; *p* = 0.751)	1.036 (0.81—1.32; *p* = 0.774)	1.143 (0.84—1.56; *p* = 0.403)	0.720 (0.33—1.58; *p* = 0.411)	1.196 (0.72—1.99; *p* = 0.492)	1.186 (0.72—1.94; *p* = 0.498)	1.058 (0.77—1.45; *p* = 0.722)	1.209 (0.77—1.89; *p* = 0.405)
	obesity (≥ 30)	0.912 (0.51—1.65; *p* = 0.760)	1.147 (0.88—1.50; *p* = 0.313)	0.935 (0.64—1.37; *p* = 0.730)	0.983 (0.43—2.25; *p* = 0.968)	1.151 (0.65—2.04; *p* = 0.631)	**1.746 (1.03—2.95;** *p*** = 0.037)**	1.116 (0.80—1.56; *p* = 0.521)	0.978 (0.59—1.61; *p* = 0.930)
**Albuminemia**	numeric value	0.989 (0.95—1.03; *p* = 0.590)	0.997 (0.97—1.03; *p* = 0.840)	1.010 (0.97—1.05; *p* = 0.642)	0.948 (0.87—1.03; *p* = 0.221)	0.971 (0.92—1.03; *p* = 0.331)	0.959 (0.92—1.00; *p* = 0.060)	0.970 (0.93—1.01; *p* = 0.147)	1.060 (0.99—1.14; *p* = 0.118)
**Smoking history**	Never_smoker	(reference)			(reference)			(reference)	
	Former_smoker	1.009 (0.69—1.47; *p* = 0.963)	1.180 (0.92—1.52; *p* = 0.199)	0.939 (0.66—1.33; *p* = 0.726)	1.465 (0.63—3.43; *p* = 0.378)	1.167 (0.68—2.00; *p* = 0.575)	1.392 (0.81—2.40; *p* = 0.233)	1.013 (0.73—1.40; *p* = 0.939)	0.777 (0.48—1.25; *p* = 0.301)
	Current_smoker	1.144 (0.79—1.65; *p* = 0.468)	1.279 (0.97—1.68; *p* = 0.079)	1.257 (0.89—1.78; *p* = 0.198)	**2.896 (1.26—6.66;** *p*** = 0.012)**	1.460 (0.81—2.65; *p* = 0.212)	**2.931 (1.68—5.11;** *p*** < 0.001)**	1.018 (0.74—1.39; *p* = 0.913)	1.188 (0.73—1.94; *p* = 0.492)
**History of cancer**	Yes (reference No)	0.711 (0.37—1.35; *p* = 0.299)	**1.522 (1.07—2.16;** *p*** = 0.018)**	**1.674 (1.07—2.63;** *p*** = 0.025)**	0.440 (0.10—1.96; *p* = 0.281)	1.631 (0.88—3.01; *p* = 0.117)	1.269 (0.68—2.38; *p* = 0.456)	0.906 (0.54—1.53; *p* = 0.710)	0.756 (0.32—1.77; *p* = 0.517)
**Stage**	0/I	(reference)			(reference)			(reference)	
	II	**3.754 (2.36—5.97;** *p*** < 0.001)**	**2.622 (1.96—3.51;** *p*** < 0.001)**	**1.696 (1.24—2.32;** *p*** = 0.001)**	1.638 (0.74—3.60; *p* = 0.219)	**1.654 (1.03—2.64;** *p*** = 0.036)**	1.263 (0.83—1.93; *p* = 0.279)	1.562 (0.99—2.47; *p* = 0.057)	**2.377 (1.21—4.68;** *p*** = 0.012)**
	III	**10.45 (6.34—17.25;** *p*** < 0.001)**	**8.416 (6.19—11.45;** *p*** < 0.001)**	**3.862 (2.64—5.66;** *p*** < 0.001)**	**2.805 (1.00—7.88;** *p*** = 0.050)**	1.506 (0.67—3.39; *p* = 0.321)	1.025 (0.46—2.28; *p* = 0.952)	**2.131 (1.33—3.41;** *p*** = 0.002)**	**3.139 (1.58—6.23;** *p*** = 0.001)**
**Histology**	Invasive ductal carcinoma	(reference)			(reference)			(reference)	
	Invasive lobular carcinoma	0.912 (0.51—1.65; *p* = 0.760)	0.901 (0.65—1.26; *p* = 0.540)	1.322 (0.90—1.94; *p* = 0.152)	0.601 (0.19—1.94; *p* = 0.394)	1.275 (0.69—2.34; *p* = 0.434)	1.584 (0.89—2.81; *p* = 0.116)	0.881 (0.56—1.39; *p* = 0.584)	1.040 (0.49—2.20; *p* = 0.917)
	Other	0.995 (0.62—1.59; *p* = 0.984)	0.838 (0.58—1.20; *p* = 0.337)	0.899 (0.55—1.46; *p* = 0.667)	0.946 (0.32—2.77; *p* = 0.919)	1.068 (0.52—2.19; *p* = 0.857)	1.487 (0.79—2.80; *p* = 0.218)	1.094 (0.71—1.69; *p* = 0.685)	1.155 (0.54—2.46; *p* = 0.707)
**Subtype**	HR+/HER2-	(reference)			(reference)			(reference)	
	HR+/HER2+	0.658 (0.34—1.28; *p* = 0.217)	0.765 (0.55—1.07; *p* = 0.120)	0.635 (0.39—1.04; *p* = 0.072)	0.613 (0.13—2.97; *p* = 0.543)	1.195 (0.53—2.69; *p* = 0.665)	1.312 (0.62—2.78; *p* = 0.476)	**0.415 (0.20—0.84;** *p*** = 0.015)**	**0.465 (0.23—0.93;** *p*** = 0.030)**
	HR-/HER2+	**2.045 (1.10—3.79;** *p*** = 0.023)**	0.675 (0.40—1.15; *p* = 0.150)	**0.165 (0.04—0.67;** *p*** = 0.012)**	NE	1.222 (0.36—4.13; *p* = 0.746)	1.788 (0.75—4.24; *p* = 0.186)	0.954 (0.52—1.75; *p* = 0.878)	0.795 (0.42—1.52; *p* = 0.488)
	HR-/HER2-	**5.826 (3.97—8.56;** *p*** < 0.001)**	1.094 (0.78—1.53; *p* = 0.600)	0.924 (0.55—1.54; *p* = 0.761)	**1.513 (0.49—4.70;** *p*** = 0.474)**	1.904 (0.91—3.97; *p* = 0.086)	1.483 (0.72—3.04; *p* = 0.281)	**2.684 (1.97—3.65;** *p*** < 0.001)**	0.616 (0.33—1.15; *p* = 0.129)
**Grade**	1	(reference)			(reference)			(reference)	
	2	**3.301 (1.01—10.76;** *p*** = 0.048)**	**3.514 (1.84—6.70;** *p*** < 0.001)**	**1.941 (1.16—3.26;** *p*** = 0.012)**	0.472 (0.19—1.18; *p* = 0.108)	0.797 (0.42—1.51; *p* = 0.484)	1.556 (0.78—3.08; *p* = 0.205)	2.905 (0.71—11.90; *p* = 0.138)	1.909 (0.21—17.18; *p* = 0.563)
	3	**6.030 (1.58—23.05;** *p*** = 0.009)**	**5.143 (2.49—10.60;** *p*** < 0.001)**	1.084 (0.51—2.28; *p* = 0.832)	0.407 (0.08—1.97; *p* = 0.263)	0.523 (0.19—1.46; *p* = 0.216)	2.014 (0.68—5.98; *p* = 0.207)	**4.375 (1.03—18.62;** *p*** = 0.046)**	2.284 (0.24—21.59; *p* = 0.470)
**Mitotic Index**	1	(reference)			(reference)			(reference)	
	2	1.150 (0.66—2.00; *p* = 0.620)	**1.395 (1.03—1.89;** *p*** = 0.032)**	1.247 (0.85—1.84; *p* = 0.262)	1.898 (0.71—5.08; *p* = 0.202)	1.520 (0.81—2.84; *p* = 0.189)	0.842 (0.43—1.65; *p* = 0.617)	0.686 (0.47—1.01; *p* = 0.057)	1.633 (0.85—3.14; *p* = 0.141)
	3	1.101 (0.58—2.09; *p* = 0.769)	1.206 (0.80—1.83; *p* = 0.376)	**2.346 (1.28—4.29;** *p*** = 0.006)**	3.033 (0.79—11.58; *p* = 0.104)	1.767 (0.66—4.72; *p* = 0.256)	0.878 (0.33—2.34; *p* = 0.795)	0.870 (0.58—1.31; *p* = 0.503)	1.094 (0.52—2.29; *p* = 0.810)

NE: not evaluable (absence of event)

For relapsing patients, the following baseline characteristics were associated with worse OS after relapse: age ≥ 70 years, concomitant comorbidities, higher tumour stage and grade at diagnosis and triple-negative phenotype.

### Impact of lymphopenia at diagnosis on surgical complications and chemotherapy compliance

The impact of lymphopenia on acute toxicities (surgery, perioperative chemotherapy and radiation if indicated) is presented in Table 5. No difference was observed in the risk of surgical complications nor in compliance with the dose intensity of chemotherapy or trastuzumab. Patients with ALC < 1.0 × 10^9^ cells/L were significantly more likely to receive granulocyte-colony stimulating factor (G-CSF), either in neoadjuvant (*P* = 0.011) or adjuvant setting (*P* = 0.04).

**Table 5:** Incidence of acute toxicity according to lymphopenia at diagnosis

	Lymphocyte count at baseline		Total	*P* value
	< 1.0 × 10^9^ /L	≥ 1.0 × 10^9^ /L		
**Any post-surgical complications**				0.798
Missing	8	69	77	
no	277 (82.9%)	8716 (83.5%)	8993 (83.4%)	
yes	57 (17.1%)	1727 (16.5%)	1784 (16.6%)	
**Impact on chemotherapy regimen (if indicated)**				
**Neoadjuvant chemotherapy: necessity of a reduction in the planned dose**				0.464
no	72 (79.1%)	1129 (82.2%)	1201 (82.0%)	
yes	19 (20.9%)	245 (17.8%)	264 (18.0%)	
**Neoadjuvant chemotherapy: use of granulocyte-colony stimulating factors**				**0.011**
no	37 (41.6%)	743 (55.4%)	780 (54.5%)	
yes	52 (58.4%)	599 (44.6%)	651 (45.5%)	
**Adjuvant chemotherapy: necessity of a reduction in the planned dose**				0.589
no	73 (84.9%)	3542 (82.7%)	3615 (82.7%)	
yes	13 (15.1%)	743 (17.3%)	756 (17.3%)	
**Adjuvant chemotherapy: use of granulocyte-colony stimulating factors**				**0.04**
no	36 (42.4%)	2214 (53.6%)	2250 (53.3%)	
yes	49 (57.6%)	1919 (46.4%)	1968 (46.7%)	
**Temporary or permanent interruption of (neo)adjuvant trastuzumab (if indicated)**				0.368
no	42 (95.5%)	1177 (91.7%)	1219 (91.8%)	
yes	2 (4.5%)	107 (8.3%)	109 (8.2%)	

## Discussion

Whereas lymphopenia was largely reported in 20–25% of patients with advanced BC [8], few data exist concerning lymphopenia at stage of early BC. In the large observational CANTO cohort, the prevalence of lymphopenia < 1.0 × 10^9^ cells/L in localized BC patients at diagnosis was 3.15%. A recent study reported a prevalence of 1.08% in a female population of healthy subjects (using an identical cut-off) [16]. This study also reported a lower prevalence of lymphopenia in cases of smoking or obesity, as observed in our cohort. ALC < 1.0 × 10^9^ cells/L was additionally more frequent for patients with ECOG-PS > 0 or hypoalbuminemia and for patients with personal history of previous neoplasia. Similar results were also recently reported by Vicente-Conesa et al. in a small cohort (*n* = 103) of European early BC, treated in neo-adjuvant setting [17] and in a larger cohort (*n* = 5,252) of Korean early BC patient reported by Kim et al. [18], underlying that this finding may be independent from pharmacogenomic or environmental considerations.

In our cohort, lymphopenia was not associated with a poorer survival. In multivariable analysis, lymphopenia impact was restricted to significant risk of early relapse during the first 2 years and does not appear to be an independent prognostic factor for survival, without or in case of relapse. The classical known tumour prognostic factors were also found associated with higher risk of early relapse: stage II and III; TNBC vs. non TNBC subtype and tumour grade > 1), which was expected in such a large cohort and served as an internal positive control. Similarly, in the Vicente Conesa et al. cohort [17], a lower baseline peripheral blood lymphocyte counts was independently associated with worse disease‑free survival in patients receiving neoadjuvant chemotherapy, with no effect observed for overall survival. Kim et al. also identified in early BC an association between baseline lymphopenia (ALC < 1.0 × 10^9^ cells/L) and poor OS, BC-specific survival and distant recurrence-free survival [18]. Although methodologies and timing of assessments differ across studies, these findings align with the hypothesis that early alterations in peripheral immunity may contribute to reduced anti‑tumour surveillance and relapse risk.

Another recent study highlighted a significant prognostic value of an ALC count when assessed in 628 early BC patients before radiotherapy. ALC < 1.7 × 10^9^ cells/L was associated with a notably lower 10-year BC-specific survival (*P* = 0.0013) and OS rate (*P* = 0.026) [19]. For nearly half of these patients (45.9%), the lymphocyte count was however assessed after adjuvant chemotherapy. The prevalence of lymphopenia was therefore greater in patients who had received chemotherapy and whose prognosis is less favourable than those who do not require perioperative chemotherapy. This approach differs markedly from that of our study, where the lymphocyte count was measured before any therapeutic intervention. This allowed for the analysis of the prognostic value of baseline lymphopenia without any interference from treatments, which are themselves influenced by the tumour and patient profiles.

Our findings do not support the use of lymphocyte count as a reliable factor to stratify early BC patients according to their risk of death, either with or without relapse. The association between lymphopenia and early relapse however deserves a specific attention. This was also reported in an earlier study evaluating 308 BC cases with 21 lymphopenic patients [20]. Lymphopenia may therefore be a marker of tumour-induced immunodeficiency and therefore of intrinsic aggressiveness, independently of other known risk factors. Several publications, evaluating blood parameters from primary BC patients, reported other quantitative [21] and functional [22, 23] alterations affecting different immune populations (NK cells, T cells, monocytes). Moreover, in 40% of primary BC cases, blood monocytes failed to differentiate in vitro into M1 macrophages with GM-CSF/IL-4 cocktail [24]. This could result from release of soluble factors in serum/plasma (TGF-β, VEGF, IL-6 or M-CSF) produced by tumour cells and associated with worse prognosis for BC patients [25–27] that may alter peripheral immune cells. Taken together, these results strongly suggest that localized BC tumours may alter immune cells in the periphery and induce a deficient immune surveillance that could then be associated with a worse prognosis.

On the other hand, lymphopenia at diagnosis may be associated with a higher level of frailty in patients, which could have had an impact on the choice of peri-operative treatments. In such a real-life cohort, treatment indications were not imposed and we cannot exclude that chemotherapy indications were made differently in the presence or absence of lymphopenia which was significantly more frequent for ECOG-PS > 0 patients, patients with hypoalbuminemia and for patients with personal history of previous neoplasia. Use of G-CSF was more frequent during neo-adjuvant and adjuvant chemotherapy in case of lymphopenia at diagnosis. That included both primary and secondary prophylaxis. Lymphopenia is known to be a risk of chemotherapy-induced febrile neutropenia [28, 29] and we can assume that G-CSF were prescribed more frequently for these patients for this reason. Baseline lymphopenia didn’t ultimately result in a lower delivered dose intensity compared with the planned treatment.

Another hypothesis to explain the association between lymphopenia and early relapse is that some patients with an ALC < 1.0 × 10^9^ cells/L could present at diagnosis an undetected metastatic disease. Patients from the prospective CANTO cohort were evaluated according to standard real-world clinical practice and no exhaustive staging was required. We cannot then exclude that some patients may present undetected micro-metastases. In this context, a recent publication reported the reduced lymphocytes counts in early BC patients presenting circulating tumour cells [30]. The presence of tumour cells in the bone marrow of BC patients has been correlated with worse prognosis [31, 32], it may suggest that occult bone marrow invading tumour cells could alter bone marrow haematopoiesis.

Baseline lymphopenia may therefore reflect a micro-metastatic disease burden that remains undetected in standard early‑stage work‑up or indicate systemic immune dysregulation or tumour‑induced immunosuppression, as supported by prior reports of quantitative and functional alterations in peripheral immune populations in early breast cancer.

Together, these biological mechanisms may provide a plausible explanation for the increased risk of early relapse observed in lymphopenic patients, even in the absence of a long‑term association with overall survival.

Future studies should include lower threshold of lymphocyte count and consider lymphocyte subpopulation. CD4^+^ T lymphocyte count < 0.45 × 10^9^ cells/L has been reported to be a negative prognostic factor for advanced cancer patients [5]. In metastatic BC patients, CD4^+^ T lymphocyte count < 0.2 × 10^9^ cells/L has additionally been associated with reduced survival [29]. In the CANTO study, lymphocyte subpopulation counts were not assessable as this data was not collected prospectively. Very few patients presented with a severe lymphopenia (< 0.70 × 10^9^ cells/L, *n* = 85, 0.78%), and the survival data of patients with lymphopenia appeared to be similar regardless of the chosen thresholds. Nevertheless, a poorer prognostic value cannot be excluded for very low lymphocyte counts, especially when considering the CD4^+^ T lymphocyte subpopulation [3, 5], even if it could not be estimated in our data.

A limitation of our study is the relatively small number of patients presenting baseline lymphopenia (*n* = 342), which reduces the statistical power of subgroup and exploratory analyses and, in particular, limits the precision of the cause-specific hazard estimates. In several strata of the competing‑risk model, the number of events was low, resulting in wide confidence intervals; these results should therefore be interpreted with appropriate caution and viewed as hypothesis‑generating. As a consequence, sample sizes for each BC subtype did not allow reliable stratified modeling. Another limitation relates to the definition of lymphopenia. Although ALC < 1.0 × 10⁹ cells/L was prespecified as the primary threshold based on prior literature, we also evaluated alternative cut-offs in exploratory analyses to assess robustness. While survival patterns were similar across thresholds, exploring multiple definitions may introduce some degree of outcome‑related bias. Nevertheless, the modelling strategy was designed to maximize robustness and clinical interpretability: the piecewise Cox approach allowed us to address violations of the proportional hazards assumption by incorporating pre-specified, clinically relevant time periods, while the multivariable models systematically included established prognostic factors. This framework ensured that the analyses remained statistically rigorous and aligned with clinical knowledge, despite the limited number of lymphopenic patients.

In conclusion, based on data from a large observational prospective cohort, lymphopenia < 1.0 × 10^9^ cells/L at diagnosis can be observed in less than 5% of early BC patients, which is lower than in metastatic setting but appear to be higher than in healthy population. Even if our data do not support the use of ALC as a relevant prognostic factor for treatment decisions in early BC patients, the association with a higher risk of early relapse warrants attention, and should be interpreted in light of potential underlying biological mechanisms, including undetected micro-metastatic disease burden or systemic immune dysregulation.

## Supplementary Information

Below is the link to the electronic supplementary material.

Supplementary Material 1

## Data Availability

The data that support the findings of this study are available from Unicancer but restrictions apply to the availability of these data, which were used under license for the current study, and so are not publicly available. Data are however available from the authors upon reasonable request and with permission of Unicancer.
